# New Detection of Locally Acquired Japanese Encephalitis Virus Using Clinical Metagenomics, New South Wales, Australia

**DOI:** 10.3201/eid2903.220632

**Published:** 2023-03

**Authors:** Joel Maamary, Susan Maddocks, Yael Barnett, Stephen Wong, Michael Rodriguez, Linda Hueston, Neisha Jeoffreys, John-Sebastian Eden, Dominic E. Dwyer, Tony Floyd, Marshall Plit, Jen Kok, Bruce Brew

**Affiliations:** St Vincent's Health Australia, Sydney, New South Wales, Australia (J. Maamary, Y. Barnett, M. Plit, B. Brew);; Centre for Infectious Diseases and Microbiology Laboratory Services, NSW Health Pathology-Institute of Clinical Pathology and Medical Research, Westmead, New South Wales, Australia (S. Maddocks, L. Hueston, N. Jeoffreys, D.E. Dwyer, J. Kok);; Sydpath, St Vincent's Health Network, Sydney (S. Wong, M. Rodriguez);; University of Sydney Institute for Infectious Diseases, Westmead Institute for Medical Research, Westmead (J.-S. Eden);; Griffith Base Hospital, Griffith, New South Wales, Australia (T. Floyd);; University of New South Wales, Sydney (M. Plit, B. Brew);; St Vincent's Centre for Applied Medical Research, Sydney (B. Brew);; The University of Notre Dame Australia, Sydney (B. Brew)

**Keywords:** Japanese encephalitis virus, Japanese encephalitis, metagenomics, meningoencephalitis, meningitis/encephalitis, neurology, virology, viruses, vector-borne infections, Australia

## Abstract

In the context of an emerging Japanese encephalitis outbreak within Australia, we describe a novel locally acquired case in New South Wales. A man in his 70s had rapidly progressive, fatal meningoencephalitis, diagnosed as caused by Japanese encephalitis virus by RNA-based metagenomic next-generation sequencing performed on postmortem brain tissue.

Japanese encephalitis virus (JEV) is a single-stranded, positive-sense, RNA flavivirus endemic to tropical regions of South and Southeast Asia and is the most common cause of vaccine-preventable encephalitis in the Asia-Pacific region (*1*). As for Murray Valley encephalitis virus and Kunjin virus, 2 other flaviviruses endemic in Australia, most JEV infections are asymptomatic, but severe meningoencephalitis can occur in up to 1% (*2*) of cases. Although incidence varies geographically, ≈100,000 cases are estimated annually worldwide, resulting in 709,000 disability-adjusted life years through severe neurologic disease complications (*3*). Nonspecific febrile illness is the typical clinical manifestation, but in severe cases, rapidly progressive neurologic deterioration, reduced consciousness, movement disorders, seizures, and coma can occur. Neuroinvasive JEV mortality can reach 30%, and major neurologic disability approaches 50% (*4*).

*Culex tritaeniorhynchus* mosquitoes are the primary vector for JEV transmission in Asia. Although previously thought absent from Australia, this species was recently detected in the Darwin and Katherine regions of the Northern Territory (*5*). The *Cx. annulirostris* mosquito, which is the primary vector for Flaviviridae transmission in Australia, has also been implicated in JEV transmission globally. JEV has previously been isolated from subspecies of *Aedes* and *Anopheles* mosquitoes, both present in Australia. Wading and water birds are the virus’s natural host; feral and domestic pigs are particularly susceptible. Transmission between pigs occurs through mucosal and microdroplet contacts, enabling disease amplification and acting as a protective reservoir for the virus. However, humans are dead-end hosts, probably because of low levels of or short-lived viremia (*6*).

In Australia, sporadic human cases and virus isolation in pigs and mosquitoes have all been confined to the tropical north of the country (*7*,*8*). Since February 2022, however, JEV has been found in 4 states in Australia (New South Wales [NSW], Victoria, Queensland, and South Australia), thousands of kilometers from previously detected cases. We describe a locally acquired case of fatal JEV meningoencephalitis in a NSW resident in January 2022. The infection was diagnosed by RNA-based metagenomic next-generation sequencing (RNA-mNGS) performed on postmortem brain tissue.

## The Case

**Figure 1:**
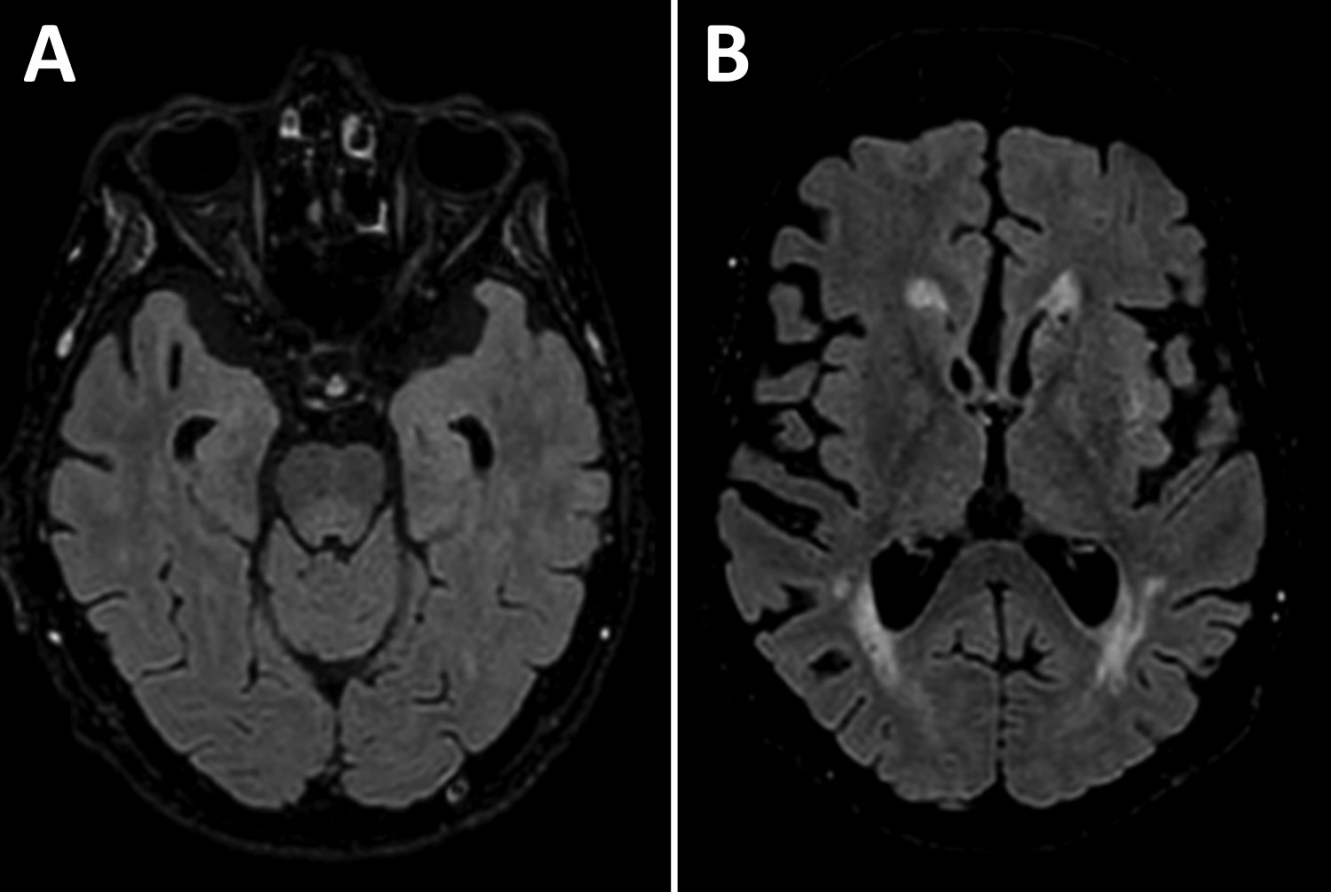
Axial FLAIR magnetic resonance imaging brain sequence in a patient with locally acquired Japanese encephalitis virus detected using clinical metagenomics, New South Wales, Australia. A) Equivocal hyperintensity in the dorsal midbrain and pons; B) sparing of the thalamus and basal ganglia.

A man in his 70s sought care at a rural hospital after 3 days of fever and progressive confusion. Results septic screen, chest radiography and computed tomography brain scan were unremarkable. Despite empiric intravenous flucloxacillin and gentamicin, the patient experienced progressive neurologic deterioration and required intubation. Cerebrospinal fluid (CSF) testing revealed a lymphocyte predominant pleocytosis of 126 × 10^6^ cells/L (50 × 10^6^ cells/L polymorphs and 76 × 10^6^ cells/L mononuclear cells) and elevated protein of 0.96 g/L (reference range 0.15–0.45). No organisms were isolated. Results of CSF nucleic acid testing (NAT) were negative for *Neisseria meningitidis*, *Streptococcus pneumoniae*, herpes simplex virus types 1 and 2, enterovirus, varicella zoster virus, parechovirus, cytomegalovirus, and Epstein-Barr virus. CSF and serum samples tested negative for antineuronal antibodies (PCA-1/PCA-2/ANNA-1/ANNA-2/Ma1/Ma2/Amphiphysin/SOX-1/CRMP-5/Tr) and limbic encephalitis panel (anti-NMDA/CASPR-2/LGI-1/GABA-B/DPPX/IgLON5/AMPA-1/AMPA-2). Empiric treatment was changed to intravenous ceftriaxone, benzylpenicillin, and aciclovir. Magnetic resonance brain imaging showed an equivocal T2/FLAIR hyperintensity in the dorsal midbrain and pons with sparing of both thalami and basal ganglia (Figure 1). Electroencephalogram demonstrated encephalopathic features. Repeat CSF examination on day 10 demonstrated lymphocytic pleocytosis (57 × 10^6^ cells/L mononuclear cells), and results of repeat culture, NAT, and antineuronal/limbic encephalitis panels were negative. CSF protein remained elevated at 0.86 g/L.

**Figure 2:**
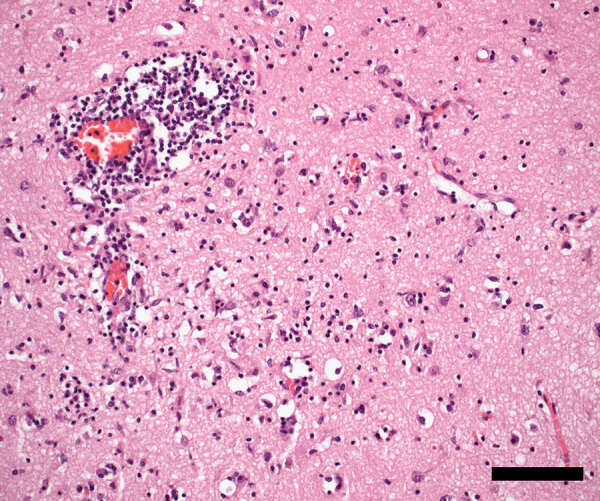
Temporal neocortex showing encephalitis with perivascular and interstitial lymphocytes, macrophages/activated microglia, and neuronophagia in patient with locally acquired Japanese encephalitis virus detected using clinical metagenomics, New South Wales, Australia. Hematoxylin and eosin stain; scale bar indicates 100 µm.

Despite supportive management and broadening of antimicrobial therapy with meropenem, the patient showed no neurologic improvement and, after discussion with his family, ventilator support was withdrawn; he died 22 days after symptom onset. In the absence of a definitive diagnosis, a noncoronial limited brain autopsy was performed. Twenty-four brain tissue samples were sent for neuropathologic examination; 10 were sent for RNA-mNGS analysis (*9*). Initial sequencing of the right anterior hippocampus, amygdala, and left striatum returned only host-derived sequences. Additional pooled samples of libraries of the left hippocampus, left upper midbrain, pons, medulla, right cerebellum, and dentate nucleus identified 4 JEV genotype IV sequences. We detected JEV RNA by real-time reverse transcription PCR targeting the JEV NS1 region (*10*) in all 10 samples (cycle threshold values 34.3–38.3). Neuropathology showed widespread meningoencephalitis, more marked in gray matter and most severe in the thalamus, hippocampus, and substantia nigra, with perivascular and interstitial lymphocytes (predominantly CD8+ T-cells), macrophages/activated microglia, and loose microglial nodules (Figure 2). Retrospective testing of stored serum samples demonstrated JEV-specific IgM and IgG seroconversion. JEV-specific IgM, but not RNA, was detected in CSF. Further history revealed the patient had recently visited a neighboring town containing numerous domestic pig farms, where JEV was detected and subsequently confirmed.

## Discussion

JEV was detected for the first time across southeastern Australia in 2022 and, as of January 5, 2023, a total of 45 human cases of JEV had been notified in Australia since January 1, 2021 (of which 14 were from NSW), including 7 deaths (*11*). The described case was undiagnosed premortem; JEV was not known to be present in NSW at the time of the patient’s admission. RNA-mNGS enabled a definitive diagnosis postmortem through the detection of JEV-specific sequences in brain tissue, confirmed by NAT and seroconversion on retrospective serum testing. The diagnosis was further corroborated by the concurrent detection of JEV in piggeries across 4 Australia states. Similar to other reported cases of infection with fatal genotype IV (*12*), this case evidenced profound neurologic deterioration 3–4 days after symptom onset with CSF lymphocyte predominant pleocytosis and JEV-specific IgM in CSF. However, unlike other case reports, some typical markers of JEV, such as T2/FLAIR hyperintense signal change in both thalami, basal ganglia, and substantia nigra, occasionally associated with hemorrhage, were not present in this case. The lack of JEV RNA detected in CSF is not uncommon because of the relatively brief viremia in humans, although prolonged viruria of 26 days and viremia of 28 days have been reported (*13*). JEV is typically diagnosed by the detection of JEV-specific IgM in CSF or through JEV-specific IgG seroconversion in serum samples, but false-positive results of serologic testing for JEV might occur because of cross-reactivity to other flaviviruses.

This case highlights the diagnostic value of pathogen-agnostic mNGS for pathogens not identified through traditional testing, known but unexpected pathogens, or novel pathogens. Recent public health alerts should prompt clinicians to interrogate a patient’s history for animal or mosquito exposures and request specific JEV or other flavivirus (such as Murray Valley encephalitis virus and Kunjin virus) testing accordingly when treating undifferentiated meningoencephalitis. The case also demonstrates the new incursion of a pathogen across a broad, previously nonendemic geographic area and into a largely nonimmune population. The origins remain unclear but, given the widespread geographic area of infected piggeries and human cases, JEV has likely been circulating undetected in wild birds, mosquitoes, and pigs for some time. Whole-genome sequencing has demonstrated that this outbreak is caused by genotype IV, previously thought to be restricted to Indonesia, Papua New Guinea, and the Tiwi Islands.

Changes in vector distribution have been associated with changes in climate, destruction of natural habitats altering bird migratory patterns, agricultural practices, and periurban growth (*14*). Floodwater-mediated or windblown movement of JEV-infected mosquito vectors into new regions has been previously reported in Australia (*15*). The movement of other infected vertebrates could also be implicated. Mosquito, human and animal surveillance in areas where JEV is detected will inform the extent of JEV incursion in mainland Australia and guide vector control, vaccination efforts, and research priorities, including vector competence studies to limit further disease and vector spread.

## References

[1] Kwak BO, Hong YJ, Kim DH. Changes in age-specific seroprevalence of Japanese encephalitis virus and impact of Japanese encephalitis vaccine in Korea. Clin Exp Pediatr. 2022;65:108–14. 10.3345/cep.2020.01984 34592804 PMC8898622

[2] Southam CM. Serological studies of encephalitis in Japan. II. Inapparent infections by Japanese B encephalitis virus. J Infect Dis. 1956;99:163–9. 10.1093/infdis/99.2.163 13376909

[3] Mathers CD, Ezzati M, Lopez AD. Measuring the burden of neglected tropical diseases: the global burden of disease framework. PLoS Negl Trop Dis. 2007;1:e114. 10.1371/journal.pntd.0000114 18060077 PMC2100367

[4] Solomon T, Dung NM, Kneen R, Gainsborough M, Vaughn DW, Khanh VT. Japanese encephalitis. J Neurol Neurosurg Psychiatry. 2000;68:405–15. 10.1136/jnnp.68.4.405 10727474 PMC1736874

[5] Lessard BD, Kurucz N, Rodriguez J, Carter J, Hardy CM. Detection of the Japanese encephalitis vector mosquito *Culex tritaeniorhynchus* in Australia using molecular diagnostics and morphology. Parasit Vectors. 2021;14:411. 10.1186/s13071-021-04911-2 34407880 PMC8371801

[6] Filgueira L, Lannes N. Review of emerging Japanese encephalitis virus: new aspects and concepts about entry into the brain and inter-cellular spreading. Pathogens. 2019;8:111. 10.3390/pathogens8030111 31357540 PMC6789543

[7] Pyke AT, Williams DT, Nisbet DJ, van den Hurk AF, Taylor CT, Johansen CA, et al. The appearance of a second genotype of Japanese encephalitis virus in the Australasian region. Am J Trop Med Hyg. 2001;65:747–53. 10.4269/ajtmh.2001.65.747 11791969

[8] Van Den Hurk AF, Montgomery BL, Northill JA, Smith IL, Zborowski P, Ritchie SA, et al. Short report: the first isolation of Japanese encephalitis virus from mosquitoes collected from mainland Australia. Am J Trop Med Hyg. 2006;75:21–5. 10.4269/ajtmh.2006.75.21 16837702

[9] Annand EJ, Horsburgh BA, Xu K, Reid PA, Poole B, de Kantzow MC, et al. Novel Hendra virus variant detected by sentinel surveillance of horses in Australia. Emerg Infect Dis. 2022;28:693–704. 10.3201/eid2803.211245 35202527 PMC8888208

[10] Shao N, Li F, Nie K, Fu SH, Zhang WJ, He Y, et al. TaqMan real-time RT-PCR assay for detecting and differentiating Japanese encephalitis virus. Biomed Environ Sci. 2018;31:208–14. 29673443 10.3967/bes2018.026

[11] Australian Government Department of Health and Aged Care. Japanese encephalitis virus (JEV) [cited 2023 Jan 24]. https://www.health.gov.au/health-alerts/japanese-encephalitis-virus-jev/japanese-encephalitis-virus-jev

[12] Pyke AT, Choong K, Moore F, Schlebusch S, Taylor C, Hewitson G, et al. A case of Japanese encephalitis with a fatal outcome in an Australian who traveled from Bali in 2019. Trop Med Infect Dis. 2020;5:133. 10.3390/tropicalmed5030133 32825150 PMC7558094

[13] Huang GKL, Tio SY, Caly L, Nicholson S, Thevarajan I, Papadakis G, et al. Prolonged detection of Japanese encephalitis virus in urine and whole blood in a returned short-term traveler. Open Forum Infect Dis. 2017;4:ofx203. 10.1093/ofid/ofx203 29226169 PMC5714136

[14] Connor B, Bunn WB. The changing epidemiology of Japanese encephalitis and New data: the implications for New recommendations for Japanese encephalitis vaccine. Trop Dis Travel Med Vaccines. 2017;3:14. 10.1186/s40794-017-0057-x 28883984 PMC5537987

[15] Ritchie SA, Rochester W. Wind-blown mosquitoes and introduction of Japanese encephalitis into Australia. Emerg Infect Dis. 2001;7:900–3. 10.3201/eid0705.017524 11747709 PMC2631883

